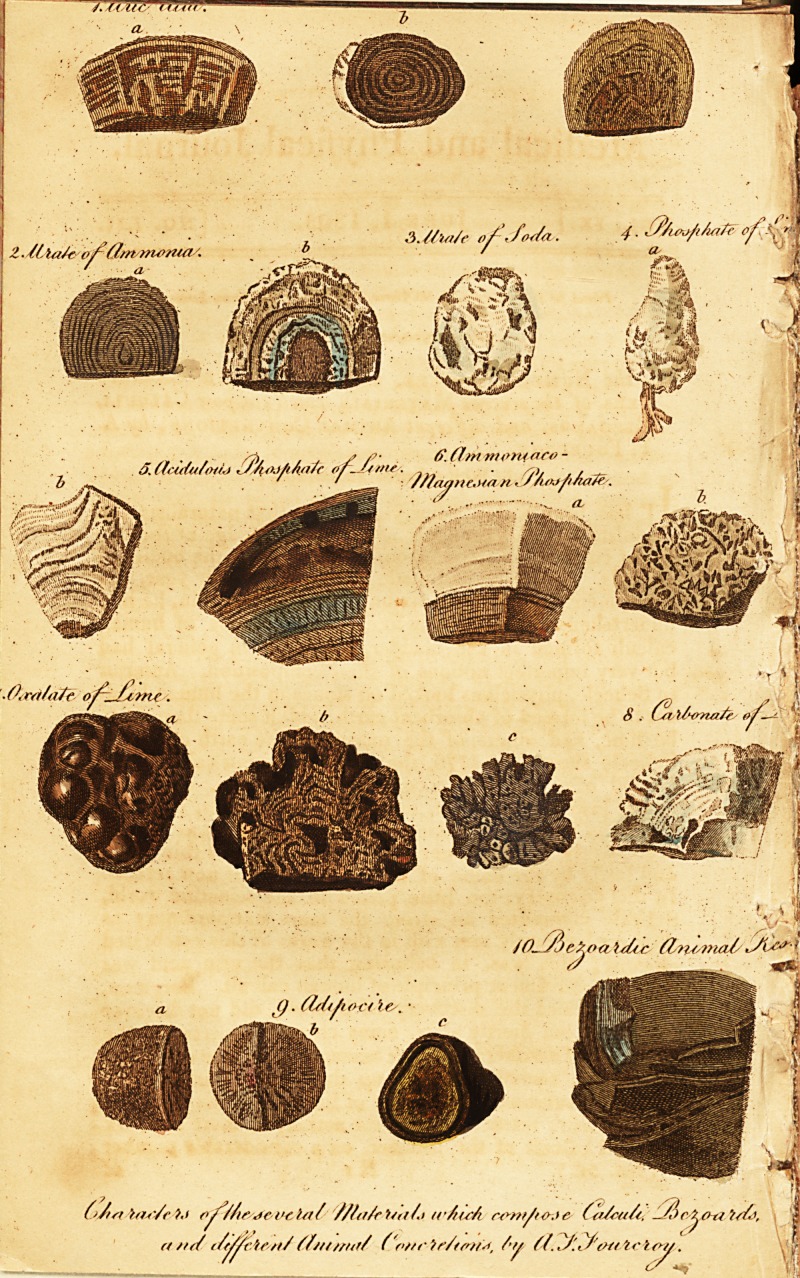# On the Number, Nature, and Distinctive Characters of the Several Materials Which Compose Calculi, Bezoards, and Different Animal Concretions

**Published:** 1803-06-01

**Authors:** A. F. Fourcroy


*Characters of the several Materials which compose Calculi, Bezoards, and different Animal Concretions, by A. F. Fourcroy.*

THE

Medical and Phyfical Journal.

VOL. IX.] JUNE 1, 1803. [NO. LII.

Printed for R. PHILLIPS, by W. Thorne, Red Lion Court, Fleet Street, London.

### *On the* NUMBER, NATURE, *and* DISTINCTIVE CHARACTERS *of the several* MATERIALS *which compose* CALCULI, BEZOARDS, *and different* ANIMAL CONCRETIONS; *by* A. F. FOURCROY.

[ With coloured Engravings. ]

IT has been within a few years only, that naturalists and chemists have acquired any accurate knowledge of the different concretions spontaneously generated in the bodies of animals. Before he had made known in the 7th year, the experiments which, in common with M. Vauquelin, he had undertaken many years before, on the nature of human calculi formed in the bladder, the world in general had but very imperfect notions of their composition. Anterior to Scheele, physicians looked on stones in the human bladder to be formed of absorbent earth, which some thought to be lime, and others of the nature of the earth of bones. Scheele shewed in 1776, that urinary human calculi were formed of a particular concrete acid, little, or rather nearly insoluble in water, or in the weaker acids, but very soluble in the fixed caustic alkalis, and which was known successively by the names of benzoardic and lithic acids, and lastly by the name of uric acid, which it now retains. In this discovery, too little prized in the scientific world, Scheele committed an error, the more extraordinary as they are but rarely met with in the works of this celebrated chemist; this was, in believing that the uric acid was the only constituent principle of human calculi. Bergman, who confirmed the discovery of Scheele, did not discover this error, but shortly after, many chemists ascertained the existence of another substance accompanying the uric acid, and which sometimes made up the greater part of their bulk; this substance is *phosphat* of lime, the basis of bones. Our extensive researches, says the author, on more than six hundred calculi of the bladder, on a considerable number of bezoards, and on a variety of other concretions found in different parts of man and animals in general, have convinced us, that besides the *uric* acid, and the *phosphat* of lime, there may be found in these concretions, *urat of ammoniac,* urat of soda, a *magnesio-ammoniacal* phosphat, an *acid phosphat* of lime, and even sometimes of *silex,* and lastly a particular grease, which the author has named *adipocire,* and a resinous substance.

To these different constituent materials is to be added a gelatinous animal substance which accompanies all the salts enumerated above, and which, with the exception of the uric acid, adipocire, and resin, makes a necessary part of all concretions, serves to unite and glew their particles together. Thus then we have twelve different substances forming animal concretions instead of two, the number supposed to exist before our author's experiments; and as they may be found in different regions, and in different animals, he thinks that they should not be in future confounded under one or two improper denominations. Besides, he remarks, that each of them exists only in particular organs, and sometimes even in particular animals. A collection of these concretions is necessarily comprehended in every cabinet, yet notwithstanding the present advanced state of chemistry, there exists on this subject an uncertainty, nay, even a disorder, which should be no longer allowed. The author has therefore conceived it of advantage to observe, with respect to these bodies, what has been already introduced into other branches of this science, namely, to establish a classification and arrangement founded on their essential characters; in a word, to preserve that clearness and precision which at present so very eminently distinguish every branch of Zoology. Chemistry, by making us acquainted with the different materials which constitute the species and the varieties of these concretions, affords us at the same time characters in each of them sufficiently well marked and contrasted to furnish distinctive characters, and in future to prevent their being confounded one with the other. The author has already remarked, that his analysis had offered him twelve different substances in those animal concretions which he has as yet examined, and though he cannot assert that nature has limited herself to these twelve, still he thinks it highly probable that it approaches nearly to the true number, or if there should be found to exist another substance, he thinks it must be extremely rare: Since six hundred human calculi of the bladder, more than fifty biliary, taken from from man and other animals, twenty-five concretions taken from other parts of the animal body, and thirty varieties of bezoards, offered but the twelve substances already enumerated. In describing the physical and chemical properties which belong to each of them, the author gives, he thinks, a sufficiently exact knowledge of their ingredients as well as a sure mode of distinguishing them.

### I. *Of the Uric Acid.*

Physical characters.—The uric acid, one of the substances most frequently found in human calculi, is disposed in dense layers,of a dusky yellow colour, the shades of which vary from that of straw to that of red marble, of rhubarb or bark. It is susceptible of a slight polish, and sometimes offers naturally this appearance. It is extremely brittle, chipping on the smallest fall or shock, it is easily reduced to powder of a pale yellow colour bordering on orange. It has neither taste nor smell. In the human kidney and bladder it is found of all sizes, from a grain of millet to that of a small melon. Its form is liable to great variety.

Chemical characters.—The uric acid turns black without melting on red cinders; it exhales a smell of burned bones, and gives carbonat and prussiat of ammoniac by distillation. It is almost insoluble in cold water; boiling water dissolves some thousand parts of its weight, but deposits almost the entire on cooling, in the form of small needle like crystals, of a paler colour than before dissolution. It is not attacked by many of the weaker acids nor by many of the more powerful; the concentrated nitric acid dissolves it by the aid of heat, and converts it into prussic and oxalic acid, and becomes of a brilliant red colour; the super oxygenated muriatic acid changes it almost immediately into malic acid. The pure fixed alkalis, potash and soda, either liquid or concentrated, soften it into a kind of soap, and dissolve it with the assistance of a little water. All the acids, even the carbonic, precipitate it from these dissolutions in form of a fine white powder. Lime, barytes, and ammoniac do not produce the same effect, because they form with it insoluble salts; the lixivia of potash and soda are therefore the real and only solvents of calculi formed of uric acid. This acid has been as yet discovered only in the human urine, and is found to exist almost in every instance; it forms the small red crystals which are found deposited on the surface of chamber-pots; it is abundantly precipitated after febrile diseases, which physicians call critical. (Vide Plate, No. 1, a, b, c.)

### II. *Urat of Ammoniac.*

Physical characters. The urat of ammoniac, the existence of which, in urinary calculi, was unknown before our discoveries, and which is sometimes found to form entirely these substances, resembles frequently in appearance those formed of uric acid, and then the dusky colour layers, similar to coffee and milk, are fine and less marbly striated or fibrous than those made up of the pure acid. For the most part, this salt is of a greyish white or pearl colour, of a pretty dense texture, smooth and brilliant in some of its layers when it has been sawed, but generally porous and cellular. It has no sensible smell.

Chemical characters. On the first impression of heat applied with the blow-pipe, the urat of ammoniac gives ammoniac which is rapidly disengaged, after which it is similar in every respect to the uric acid. It is not sensibly soluble in water, but by an access of ammoniac it becomes so; all the acids, even the weakest, take from it its ammoniac and leaves its acid in a pure state. The fixed caustic alkalis immediately disengage its ammoniac, the smell of which characterises the urate of amm. (Vide No. 2, a, b.)

### III. *Uriat of Soda.*

The combination of uric acid and soda was first announced by Mr. Tennant, as forming gouty concretions. We have twice met with this matter in our analyses; the following are its characters:

Physical characters. It is often under the form of irregular fragments glued together, without any arrangement of crystallization; its colour is white, without any polish, and it is made up of large grains, which lead one to think that it had been deposited rapidly. It is without smell, has a maukish taste, and has but little consistence. Though it is not absolutely friable, the coherence of its particles resembles that of vegetable matter, something like mushroom, or agaric, it is therefore compressible. When divided by a cutting instrument the surface shines, at least where the particles are most compact.

Chemical characters. The urat of soda, though not fusible in hot coal, or by the blow-pipe, still becomes quickly black and carbonifies. It exhales a thick fœtid smoke, as if from burned flesh. The carbon which results is of a deep black, and of but small volume. When it has been strongly calcined and washed in water, the water contains a carbonat and a prussiat of soda. It is not sensibly soluble luble in water, but when boiled in it a long time it gives it a soapy appearance, a thick froth is thrown on the surface, which has a taste similar to boiled tendons or ligaments; in this way a gelatinous substance is extracted, and which seems to make a great part of this concretion; caustic alkali renders it soluble. The acids take from it its soda, and leaves the uric acid free. This combination has been found only in depositions of gouty matter in the articulations; and I have been able to procure but two of these during ten years that I have been occupied on this subject. (Vide No. 3.)

IV. *Phosphat of Lime.*

Physical characters. It is found in three different states in those concretions of which it forms the base; it is some times hard, bonelike, and granulated; of a greyish or yel low colour, and, like bone, susceptible of a true polish Such are the protruded stones of the pineal gland, of the lachrymal, salival, bronchial glands, &c. Sometimes it is formed of thin concentric layers, of an unpolished white colour, and easily separated as in urinary calculi; portions of human urinary calculi are of this nature. It has sometimes the compact hardness of ivory, giving out the same smell when sawed, and susceptible of very fine polish; it is found in this state in some ossifications of the soft parts, and in some variety of bezoards.

Chemical properties. The phosphat of lime has properties which distinguish it from every other kind of calculus; under the blow-pipe it becomes black, and exhales an animal odour; it at length becomes white and brittle; it is not attacked by the alkali; it is indissoluble in sulphuric acid, but very much so in the nitric and muriatic acids. These dissolutions are precipitated by the alkalis and even by ammonia, and the precipitate is always a phosphat of lime; they also give a calcareous oxalat, insoluble by the oxalat of ammonia or by the oxalic aciduli. Thus it is that we discover the presence of chalk. With respect to the phosphoric acid, we are assured of its existence by adding sulphuric acid; on decanting the liquid portion which swims on the top of the sulphat of lime on evaporating this liquor, it swells, becomes as thick as honey, melts into vitreous globules, and gives phosphorus with charcoal. These trials may even be made with the blowpipe. (Vide No. 4, a, b.)

V. *Acid Phosphat of Lime.*

Physical characters. In bezoards, which it principally composes, it is found in smooth layers, striated in its fractures, and but little adherent to each other. These layers are brittle and variable as to thickness, and remarkable for shades of green or grey colour. Its taste is rather sharp and acid; the smallest effort breaks it, and its fractures are remarkable for a brilliant, granulated, and crystalline appearance.

Chemical characters. It is fusible by the blow-pipe, gives out a slightly aromatic smell as it rises, forms vitreous globules, white and opake, and gives out a phosphoric light when perfectly red. It is soluble in cold water even more than in hot, in which it separates in the form of small, brilliant spangles as it cools. It is affected by the acids in the same way as the phosphat of lime, and the alkalis change it immediately into this salt by taking from it a portion of its superabundant acid. Cit. Fourcroy has as yet found it only in the bezoards of some of the mammali. (Vide No. 5.)

VI. *Ammoniaco-Magnesian Phosphat.*

Physical characters. This substance is easiest known and best characterised; yet, notwithstanding these descriptive marks, its existence was unknown. It is generally found under two forms, sometimes in real crystals of a prismatic shape and semitransparent whitish colour; sometimes it is found flattened with rising edges, and running over the surface of animal concretions, as may be seen in some human calculi in the bezoards found in the intestines of horses, of the elephant, &c. Sometimes it is found in spathic layers, semitransparent, of different thickness, and covering the primitive nucleus of uric acid or some other matter; these layers imitate so closely calcareous spath, that Daubenton and Vicq. d'Ayzr, who first described them, have universally confounded them with this fossil. The ammonaico-magnesian phosphat is easily reduced to the state of a white powder, light, and resembling magnesia or flour, having a mawkishly sweet taste, but without that dryness which characterises phosphat of lime.

Chemical Characters. These are no less remarkable than its physical. It is blackened by the action of the blow-pipe on charcoal, and exhales a slight animal smell, which afterwards is changed to an ammoniacal. It is melted by a considerable heat; it is soluble in hot water, and crystallizes on cooling. Fixed alkalis disengage the ammonia, and separate the magnesia and form alkaline phosphats easily crystallizable. Acids dissolve it readily, without without any effervescence. When kept in pretty large pieces in weak muriatic acid, it is found after some hours like membranous flocculi, light and transparent, more abundant, and approaching nearer to the primitive form of the calculous fragments than is observed to be the case with phosphat of lime, which presents the same character but to a much less degree. It is often found to form the exterior layer of human urinary calculi. It constitutes the greater part of those bezoards found in the intestines of the horse, elephant, and larger mammiferous animals. It is never perceived in the bezoards found in the bladder of the same animals. As well as in human urinary calculi, its existence has been proved in the human urine, first as a phosphat of magnesia, and afterwards combined with ammoniac, and which is formed when the urine begins to change sufficiently to produce ammoniac. This triple salt is precipitated in the form of hexaedral prisms on the surface of glass vessels in which it had been kept, and under the pellicle which this fluid often forms on its surface. (Vide No. 6, a, b.)

VII. *Oxalat of Lime.*

Physical characters. We should hardly expect to find this substance among the constituent principles of animal concretions; and the author tells us, that it has often escaped his analysis. On one occasion, however, he ascertained its physical properties sufficiently; and he has constantly found it in that species of human calculus called by Surgeons the mulberry stone, so termed from its colour and exterior appearance. This salt, in the animal body, is of a dusky brown colour, exteriorly unequal in its surface, and beset with tubercles, which are sometimes pointed and sharp. Interiorly it is extremely hard and difficult to cut, and gives out by the friction of the saw, a mawkish animal smell, presenting a finely polished surface of a pearl colour, and formed by concentric layers.

Chemical characters. The oxalat of lime, heated with the blow-pipe, gives a smell resembling burned leather; it turns black, and gives out much smoke; it is easily converted into cinder, and leaves a white residuum soluble in water, and shewing all the characters of quick lime. It was this circumstance which manifested the existence of a calcareous salt, the acid of which was dissipated by heat, that first led the author to its discovery. It is soluble in water, which takes from it however, on boiling, a quantity of gelatinous matter. The weaker acids do not affect

 it.

it. Nitric acid, rather strong, dissolves it. Alkalis do not act on it; but carbonats of pot-ash or soda decompose it in the moist way; and this is the only process by which the author was enabled to demonstrate at once the existence of oxalic acid and lime. In this case there is formed a carbonat of lime which remains at the bottom of the liquor, and an alkaline oxalat which is dissolved. This compound has been as yet found only in the human urinary calculus. (Vide No. 7, a, b, c.)

### VIII. *Carbonat of Lime.*

Physical characters. This substance, so long known in medical books to be the only basis of those concretions found in man and other animals, has not been as yet perceived in a crystalline form, nor in that compact lamellated state in which other concretions are found. Its form is extremely irregular, sometimes however it is met with oval or rounded, or made up of an aggregation of particles tied together by animal gluten. It is for the most part white or grey, but sometimes of a dusky yellow colour.

Chemical properties. Two characters distinguish it from every other animal concretion. It gives quick lime after complete calcination, and effervesces with nitric and muriatic acids, in which it is readily dissolved. The animal matter which is more or less abundant is dissolved in boiling water and destroys its consistence. It becomes brittle when submitted to fire in Papin's digestor, like calcareous phosphats and oxalats. This substance has never been found in the concretions of the human body. It is in the bladder and kidneys of mammiferous animals that it is met with, especially in the horse, ox, and hog. (Vide No. 8.)

### IX. *Silex.*

Silex is the more rarely found of any of the substances enumerated in the composition of animal concretions. The author found it only in two of the six hundred of which he gives the result of his analysis. In these, the silex was found mixed with four other different materials, and which was only detected in the residuum after the process was finished. Its insolubility and hardness, and the manner it acted on metal blades, left no room to doubt of its presence, and will serve in future to characterize it. It is so seldom met with in concretions, and is so small in quantity, that it should be regarded as the effect of chance. The author has given no engraving of the calculi in which he found it.

X. *Adipocire.*

### X. *Adipocire.*

The author has, for many years, applied this term to a particular oily concrete found in different animal compounds, and the nature of which is between grease and wax. It is analogous to spermaceti, although it differs from this too by being less dry and more fusible; but the analogy induced the author to comprehend this last under the generic name of Adipocire. All the soft parts of animals have a singular tendency to run into this state by the putrefactive process; but the author having found it in many other cases of animal combination, and as he also found it to possess qualities according to the different circumstances of its formation, and the place it occupied, he speaks here only of that state in which it is found in the gall bladder of the human subject.

Physical characters. It is often found composed of brilliant lamulæ, white and pure, or else, as in the concretion already cited, covered with a brown coloured matter. Sometimes it is found in small striæ traversing these concretions; it is soft and greasy when pressed between the fingers; when rubbed or heated it has a maukish smell, approaching much to that of tallow or spermaceti; it is very light, and floats on water.

Chemical characters. Like spermaceti, it is melted by the same degree of heat. When melted it appears like a yellow oil; like wax, it is reduced to vapour, and sublimates above the temperature necessary to melt it. It yields water, sebacic and acetous acids, also hydrogenous carbonated gas by distillation. Its fusibility is such as renders it less capable of decomposition by an open fire than grease. It is but little affected by acids; it unites readily with alkalis, with which it forms a soap; it is insoluble in water, and soluble in alcohol, more when heated than when cold, so that it is separated from it on cooling in the form of brilliant crystals. Water precipitates from its dissolution in alcohol, and in this property resembles camphor; it dissolves in the fixed oils, and even in the volatile oils, by the addition of heat. It has been, as yet, found only in the human gall bladder. It is sometimes pure and isolated in those that are white. It is not found in the biliary concretions of the ox, nor in those of any other animal with which the author is acquainted. (Vide No. 9, a, b, c.)

XI. *Animal*

### XI. *Animal Bezoardic Resin.*

History. There exist animal concretions, either in the whole or in part resinous, which have been as yet but imperfectly made known, and then even on the report of their possessing virtues almost miraculous. The excellence of bezoardic medicines has been extolled not a century back. Such were the concretions, probably intestinal and biliary, formerly found in the Materia Medica, under the title of Eastern Bezoards. We must take care not to confound them with the hard eastern bezoards, and apparently earthy, formed of carbonat of lime, an acid phosphat of lime, or an ammoniaco-magnesian phosphat.

Physical characters. Its extreme surface is smooth and polished as marble; of a deep green, sometimes a brown colour, sometimes veined like marble, and frequently exhales, when rubbed, a sharp aromatic smell. It is easily broken, and separates into brittle laminas of a more or less darker colour than the outer layer. These layers, which are concentric, are generally of equal thickness down to the centre or nucleus, which is frequently found to be a cherry-stone, and which proves it to have been intestinal.

Chemical characters. Although its analysis has not been pushed very far, yet we know enough to distinguish animal resins from the preceding substances. The matter of bezoards softens and melts by heat. A heated needle passes through it readily. It gives out a strong aromatic musk-like smell; inflames and burns with a thick smoke. Although not soluble in boiling water, it gives to it a colour. Alcohol dissolves it entirely, and becomes coloured; this is precipitated by the addition of water in the form of a paint. Caustic alkalis dissolve it; in this it differs from vegetable resins. We can perceive therefore why the eastern bezoards were formerly imitated and made, by melting and mixing together a variety of resins, and of gum resins, to which amber and musk were added as well as gold leaf. The Goa stone of the Materia Medica is a factitious bezoard of this kind. There is also a resinous matter, less fine and less compact than the foregoing, in some human biliary concretions, and which is sometimes found in the gall bladder of the ox; this last is used in painting. In the elephant that died lately in the menagerie in Paris, and which was dissected by C. Cuvier, a stone of this kind was found and sent to the author.

XII. *Gelatine.*

### XII. *Gelatine.*

The author makes this the last and twelfth substance of animal concretions, which, for the most part, accompanies these bodies in their formation, particularly the earthy phosphats, the carbonat of lime, the oxalat of lime, and uriat of soda. This animal matter, which seems to resemble gelatine, is never found alone, and, in fact, cannot alone form calculous concretions, since it never takes on that solid concrete state which characterises these bodies. It gives however to the other substances the consistence, the bond of union or cohesion, which distinguishes them; in short, it glues their particles together. It should therefore be counted among the constituent parts of calculous concretions, and its presence is always marked by the fetid smell which it gives out on burning; by its being changed into coal by fire; by the animal smell which it imparts to water in which it has been boiled, and by its being precipitated by tannin. Its presence the author conceives to be an incontrovertible proof of the animal origin of concretions, and consequently forms one of the most certain characters of this kind of natural productions. The author thinks that this animal gluten of calculi varies in its nature, or is not always the same in different kinds of concretions.

## Figures and Tables

**Figure f1:**